# *Angiotensin I converting enzyme* gene polymorphisms and risk of psychiatric disorders

**DOI:** 10.1186/s12888-022-04007-w

**Published:** 2022-05-23

**Authors:** Mohammadarian Akbari, Reyhane Eghtedarian, Bashdar Mahmud Hussen, Solat Eslami, Mohammad Taheri, Soudeh Ghafouri-Fard

**Affiliations:** 1 Skull Base Research Center, Loghman Hakim Hospital, Shahid Beheshti University of Medical Sciences, Tehran, Iran; 2 Phytochemistry Research Center, Shahid Beheshti University of Medical Sciences, Tehran, Iran; 3 Department of Pharmacognosy, College of Pharmacy, Hawler Medical University, Kurdistan Region, Iraq; 4 Dietary Supplements and Probiotic Research Center, Alborz University of Medical Sciences, Karaj, Iran; 5 Department of Medical Biotechnology, School of Medicine, Alborz University of Medical Sciences, Karaj, Iran; 6 Men’s Health and Reproductive Health Research Center, Shahid Beheshti University of Medical Sciences, Tehran, Iran; 7 Department of Medical Genetics, School of Medicine, Shahid Beheshti University of Medical Sciences, Tehran, Iran

**Keywords:** ACE, Polymorphism, Bipolar disorder, Schizophrenia, Obsessive–compulsive disorder

## Abstract

Angiotensin-converting enzyme (ACE) as an important enzyme in the renin-angiotensin system facilitates biogenesis of the functionally active product angiotensin II from angiotensin I. *ACE* gene contains a number of functional polymorphisms which modulate activity of the encoded protein. In the current case–control study, we appraised the association between the rs4359 and rs1799752 polymorphisms and risk of bipolar disorder (type I and type II; BPDI and BPDII), schizophrenia (SCZ) and obsessive–compulsive disorder (OCD). The rs4359 was associated with risk of OCD, BPDI and BPDII in co-dominant and dominant models. The rs1799752 was associated with all assessed psychiatric conditions in four inheritance models except for BPDII whose association was not significant in recessive model. The I allele of rs1799752 was associated with OCD (adjusted FDR q-Value = 4.04E-04), SCZ (adjusted FDR q-Value = 6.00E-06), BPDI (adjusted FDR q-Value = 8.40E-03) and BPDII (adjusted FDR q-Value = 6.00E-06). The effective T allele of rs4359 showed a significant association with disease risk for BPDII group. The estimated haplotypes of these polymorphisms have been distributed differently among patients and controls. Taken together, *ACE* polymorphisms can be regarded as risk factors for a variety of psychiatric disorders.

**Supplementary Information:**

The online version contains supplementary material available at 10.1186/s12888-022-04007-w.

## Introduction

Angiotensin-converting enzyme (ACE) is a key enzyme in the renin-angiotensin system that catalyzes the biogenesis of the utmost functionally active product angiotensin II from angiotensin I [1]. Two isozymes are encoded by the *ACE* gene [2]. While expression of the somatic one has been detected in several tissues, particularly lung, kidney and testicular Leydig cells, the germinal isozyme has a restricted pattern of expression. Notably, ACE enzyme is expressed in the brain tissue, where it participates in local renin-angiotensin system as well as conversion of Aβ42 form of beta amyloid to the less toxic form Aβ40. The latter function is mainly exerted by the N domain part of this enzyme [3]. As a peptide hormone, angiotensin II stimulates production of pro-inflammatory cytokines and interferes with the activity of hypothalamic–pituitary–adrenal axis in stress condition [4]. Changes in the central activity of ACE have been reported in neuropsychiatric disorders [5–7]. Particularly, interactions of angiotensin II with central dopamine can participate in the etiopathogenesis of schizophrenia [8]. Moreover, substance P as a neuropeptide substrate for ACE [9] may contribute in the etiology of psychiatric disorders such as schizophrenia (SCZ) and bipolar disorder (BPD) [10]. Substance P is a peptide mostly produced by neurons and participates in several cellular processes, such as nociception and inflammatory reactions [11]. The involvement of angiotensin II in anxiety disorder has been verified by several studies indicating its role in modulation of hypothalamic–pituitary–adrenal and sympatho-adrenal axes [12]. Besides, a recent study has reduced plasma concentration of ACE in patients with BPD [13]. Variants in *ACE* gene has been shown to be associated with both secretion of cortisol and depression during late-life [14].

Taken together, ACE can be regarded as a participant in the etiopathogenesis of different psychiatric disorders, including BPD, SCZ and the anxiety disorder obsessive–compulsive disorder (OCD) through modulation of brain function or immune responses. *ACE* gene has a number of putative functional polymorphisms among them are the single nucleotide polymorphism rs4359 and the insertion/deletion (I/D) polymorphism rs1799752.

The functionality of the rs4359 polymorphism has been deduced from its role in modulation of response of patients to the ACE inhibitor drug ramipril [15]. The rs1799752 has been shown to modulate the protective impact of renin-angiotensin system blockade in IgA nephropathy [16]. Moreover, the rs1799752 has been suggested to affect suicide attempt probably via modulating the severity of depression [17].

Based on the above-mentioned evidence, we designed the current case–control study to appraise the association between the rs4359 and rs1799752 polymorphisms and risk of BPD (type I and type II), SCZ and OCD. We hypothesized that mentioned polymorphisms can affect risk of BPD, SCZ or OCD in Iranian population.

## Materials and methods

### Patients and controls

The current study included 146 BPD type II (89 females and 57 males), 102 BPD type I patients (61 females and 41 males), 150 patients with SCZ (58 females and 92 males) and 120 OCD cases. Moreover, 319 healthy persons (80 females and 239 males) were enlisted as control subjects. All cases and controls were Persians. Blood samples were obtained from all cases and controls. Cases were diagnosed based on the Diagnostic and Statistical Manual of Mental Disorders (Fifth edition) [18]. The presence of any structural brain disorder or systemic diseases with psychiatric involvement was considered as exclusion criteria. The inclusion criteria were compliance with the mentioned diagnostic criteria and patients' willingness for participation in the study. Written informed consent forms were signed by all participants. The study protocol was approved by the ethics committees of Shahid Beheshti Universities of Medical Sciences (IR.SBMU.RETECH.REC.1400.320).

### Genotyping

The rs4359 genotypes were identified using tetra-primer amplification-refractory mutation system (ARMS)-PCR method in a similar manner to our former study [19]. Specific primers for ARMS-PCR were designed using Primer1 tool. The following primers were used: Forward inner primer (T allele): GGGTCAGACAGAACTGGGTTCAATCT, Reverse inner primer (C allele): TTCTCTAGGAAACAAAGTAATGGAGACTGG, forward outer primer: TGGCTAATGGTTACCTGACCTTGGTTAA and Reverse outer primer: TAGAGAGTGATGAATAGTGGGGTCCTGG. Annealing temperature was set in 62 °C.

Two rounds of PCR and subsequent gel electrophoresis were used for genotyping of the rs1799752. The following primers were used for the first round of PCR: TGGAGAGCCACTCCCATCCTTTCT and GACGTGGCCATCACATTCGTCAGAT. For the second round, we used Forward primer 2: TGTAAGCCACTGCTGGAGAG and Reverse primer 2: TGGCCATCACATTCGTCAGA. The PCR program comprised a preliminary denaturing phase at 95 °C for 5 min; 35 cycles at 95 °C for 30 s, specific annealing temperature for 30 s and extension at 72 °C for 60 s; and a final incubation at 72 °C for 5 min.

### Statistical methods

Statistical assessments were performed using the Statistical Package for the Social Sciences (SPSS) v.22.0 (SPSS Inc., Chicago, IL) and SNP Analyzer 2.0. Methods and tests were similar to our recently published article [20]. Frequencies of alleles and genotypes were compared between study groups using the chi-squared test. Relative risk (odds ratio (OR)) for effect alleles and genotypes was calculated using the logistic regression method. Adjusted relative risks were calculated using gender as a covariate. Associations between genomic variants and risk of psychiatric disorders were appraised in codominant, dominant, recessive and over-dominant models. The results of association analysis were described as OR and 95% confidence interval of OR (95% CI), P value and FDR adjusted q-values. The FDR adjusted q-values were measured using a stack of P values in column analyses by GraphPad Prism version 9.0. P values less than 0.05 were regarded as statistically significant. Accordance of genotype distributions with Hardy–Weinberg equilibrium, haplotype estimation and linkage disequilibrium (LD) blocking were assessed using SNP Analyzer 2.0. Association of psychiatric disorders with haplotypes was investigated using a haplotype-specific test with one degree-of-freedom. D′ and r parameters were measured for appraisal of linkage between rs4359 and rs1799752 polymorphisms. Graphics were plotted using GraphPad Prism version 9.0 for Windows (GraphPad Software, La Jolla California USA).

## Results

### General demographic data of patients and controls

The study included 102 BPD I patients (61 females and 41 males, mean age ± SD: 41 ± 3.2), 146 BPD II patients (89 females and 57 males, mean age ± SD: 44 ± 8.9), 116 SCZ patients (58 females and 92 males, mean age ± SD: 49 ± 1.0) and 120 OCD cases (87 females and 33 males, mean age ± SD: 43 ± 5.6). Moreover, a total of 319 healthy subjects (80 females and 239 males, mean age ± SD: 42 ± 2.1) were recruited as controls.

### General information of selected polymorphisms

The rs4359 (T/C) is an intronic polymorphism located at Chr 17: 63,494,982. The deletion/insertion polymorphism is also an intronic polymorphism at Chr 17: 63,488,530–63,488,543.

### Accordance with Hardy–Weinberg equilibrium

We assessed accordance of genotype frequencies of polymorphisms with Hardy–Weinberg equilibrium (Table 1). In control group, both polymorphisms were in accordance with this supposition.

**Table 1:** The results of exact test for Hardy–Weinberg equilibrium (*P* values and genotype distributions are shown)

Variants	rs4359				rs1799752			
	CC	CT	TT	P-value	DD	ID	II	*P*-value
OCD Patients	35	70	15	0.028	2	103	15	< 0.001
SCZ Patients	58	69	23	0.74	2	126	22	< 0.001
BPDI Patients	29	59	14	0.06	1	91	9	< 0.001
BPDII Patients	42	83	21	0.051	3	117	26	< 0.001
Normal controls	139	132	45	0.13	114	138	64	0.06

T/T homozygous reference; T/C, heterozygous; C/C, homozygous mutant; T, wild allele; C, mutant allele (based on SNP database); D/D homozygous reference; D/I, heterozygous; I/I, homozygous mutant; D, wild allele; I, mutant allele (based on SNP database); *OCD* (obsessive–compulsive disorder), *SCZ* (Schizophrenia), *BPDI* (bipolar disorder class I), *BPDII* (bipolar disorder class II)

### Association between polymorphisms and psychiatric disorders

The rs4359 was associated with risk of OCD (χ2 = 10.1, *P* value = 0.006), BPD I (χ2 = 9.01, *P* value = 0.011) and BPD II traits (χ2 = 10.78, *P* value = 0.005) in genotypes analyses. Moreover, this polymorphism was associated with BPD II in allelic analyses (χ2 = 5.02, *P* value = 0.025) (Table S1).

The rs1799752 was associated with risk of OCD, SCZ, BPDI and BPDII traits in both genotype and allelic analyses (Table S2).

**Fig. 1:**
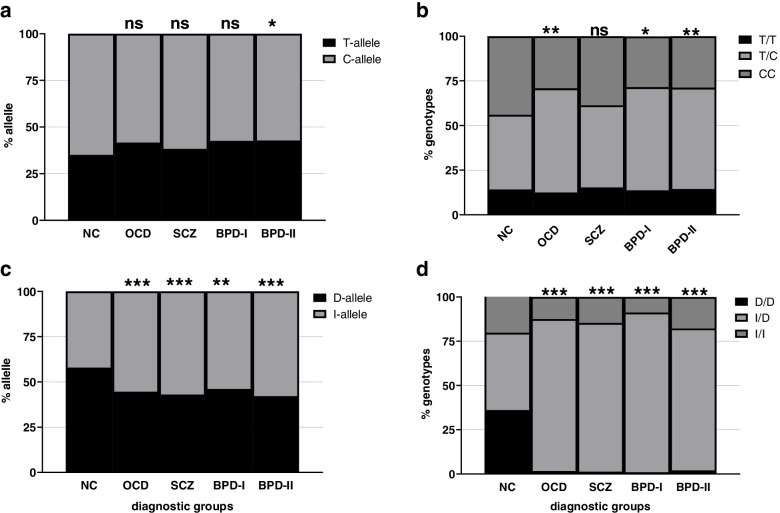
Distribution of alleles **a**, **c** and genotypes **b**, **d** of rs4359 and rs1799752 among patients with OCD (obsessive–compulsive disorder), SCZ (Schizophrenia), BPD (bipolar disorder (class I and II)) and NC (normal controls). Chi-squared test was applied to examine the differences in allele frequency and genotype distribution in patients compared to control group. (* *P* value < 0.05, ** *P* value < 0.001, **** *P* value < 0.0001 and ns; non-significant)

Figure 1 shows the distribution of alleles and genotypes of these two polymorphisms among study groups.

**Fig. 2:**
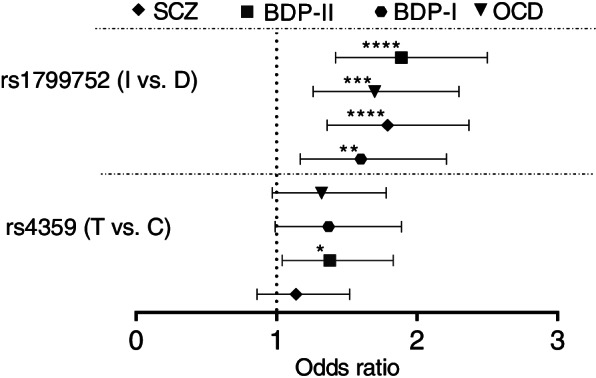
Allele model. The results of risk association for rs4359 and rs1799752 alleles. The effective alleles (T and I) were tested against C and D alleles. The Odds Ratios (plus Confidence Intervals) are reported on the X axis in a linear scale. The effective I allele showed a significant association with disease risk for all diagnostic groups. The effective T allele showed a significant association with disease risk for BPDII group. * indicates the significant results from our analyses (*p < 0.05, **p < 0.01, ***p < 0.001, ****p < 0.0001)

Then, we assessed associations between two polymorphisms and mentioned disorders in allelic model. The I allele of rs1799752 was associated with OCD (adjusted FDR q-Value = 4.04E-04), SCZ (adjusted FDR q-Value = 6.00E-06), BPDI (adjusted FDR q-Value = 8.40E-03) and BPD II (adjusted FDR q-Value = 6.00E-06) (Table 2 and Fig. 2).

**Table 2:** Risk association of rs4359 and rs1799752 in allelic model for diagnostic groups of OCD, SCZ, BPD (class I and II) with the smallest p-value (*shows significance)

Diagnostic groups	Minor Allele	OR (95% CI) (1)	*P*-Value (1)	FDR q-Value (1)	OR (95% CI) (2)	*P*-Value (2)	FDR q-Value (2)
OCD patients	T (T vs. C)	1.32 (0.97–1.78)	7.40E-02	6.14E-02	1.21 (0.86–1.67)	2.60E-01	3.15E-01
	I (I vs. D)	1.7 (1.26–2.3)	4.00E-04*	8.10E-05*	1.68 (1.21–2.34)	2.00E-03*	4.04E-04*
SCZ Patients	T (T vs. C)	1.14 (0.86–1.52)	3.40E-01	4.70E-01	1.12 (0.84–1.49)	4.20E-01	6.70E-01
	I (I vs. D)	1.79 (1.36–2.37)	3.00E-05*	6.00E-06*	1.8 (1.36–2.38)	3.00E-05*	6.00E-06*
BPD-I Patients	T (T vs. C)	1.37 (0.99–1.89)	5.30E-02	6.70E-02	1.27 (0.9–1.78)	1.60E-01	2.01E-01
	I (I vs. D)	1.6 (1.17–2.21)	3.00E-03*	3.70E-03*	1.57 (1.12–2.19)	8.00E-03*	8.40E-03*
BPD-II Patients	T (T vs. C)	1.38 (1.04–1.83)	2.50E-02	5.00E-03*	1.3 (0.96–1.76)	8.80E-02	5.54E-02
	I (I vs. D)	1.89 (1.42–2.5)	9.00E-06*	2.00E-06*	1.89 (1.4–2.5)	2.80E-05*	6.00E-06*

(1) Unadjusted, (2) adjusted by sex. Minor allele is the effect allele. *OR* Odds ratio, *FDR* false discovery rate

**Fig. 3:**
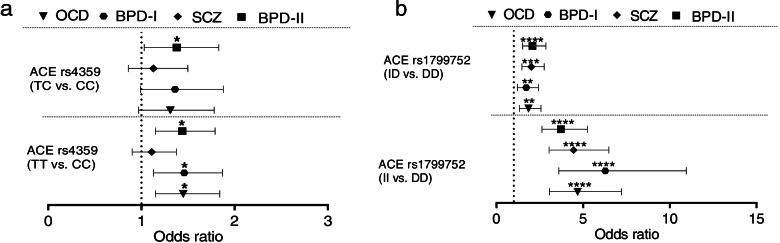
Co-dominant model. The results of risk association for rs4359 and rs1799752 genotypes. The effective genotypes TT, TC **a** and II, ID **b** were tested against CC and DD genotypes, respectively. The Odds Ratios (plus Confidence Intervals) are reported on the X axis in a linear scale. The effective II and ID genotypes vs. DD genotype showed significant effects for all diagnostic groups. * indicates the significant results from our analyses (* *p* < 0.05, **p < 0.01, ***p < 0.001, ****p < 0.0001). *OCD* Obsessive–compulsive disorder, *SCZ* Schizophrenia, *BPD* bipolar disorder (class I and II)

Then, the associations between mentioned polymorphisms and disorders were assessed in co-dominant, dominant, recessive and over-dominant models (Tables S3 and S4). The rs4359 was associated with risk of OCD, BPDI and BPDII in co-dominant and dominant models. The rs1799752 was associated with all assessed psychiatric conditions in four inheritance models except for BPDII whose association was not significant in recessive model. In co-dominant model, the effective II and ID genotypes showed significant effects as risk factors for all study groups vs. DD genotype (Fig. 3).

**Fig. 4:**
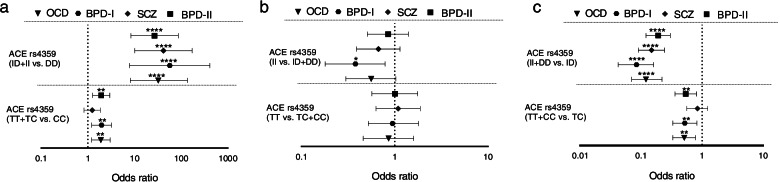
The results of risk association for rs4359 and rs1799752 genotypes by dominant model **a**, recessive model **b** and over-dominant model **c**. The Odds Ratios (plus Confidence Intervals) are reported on the X axis in a logarithmic scale. Data on the right of Y axis indicates effects toward the risk and the data on the left indicates protective effects. The effective genotypes in dominant model (a) showed significant effects toward the risk for all diagnostic groups. In this model the presence of at least one mutated (-) allele was tested against the homozygous wildtype genotype (wt/wt). The effective genotypes in over-dominant model (c) showed a significant protective effect against the risk for all diagnostic groups. * indicates the significant results from our analyses * indicates the significant results from our analyses (* *p* < 0.05, **p < 0.01, ***p < 0.001, ****p < 0.0001). *OCD* Obsessive–compulsive disorder, *SCZ* Schizophrenia, *BPD* bipolar disorder (class I and II)

The effective genotypes in dominant model showed significant association with the risk of disorder for all study groups. In this model the presence of at least one mutated allele was tested against the homozygous wildtype genotype (wt/wt). The effective genotypes in over-dominant model showed a significant protective effect against the risk for all diagnostic groups (Fig. 4).

## Haplotype analyses

Based on the calculated D and r values, the assessed polymorphisms within ACE gene were not in the strong LD. All estimated haplotypes except for T I haplotype were differently distributed among OCD cases and controls. Moreover, C D and C I haplotypes were associated with decreased and increased risk of SCZ, respectively. However, these haplotypes were associated with increased and decreased risk of both BPDI and BPDII, respectively. Table 3 shows the distribution of estimated haplotypes between study groups.

**Table 3:** The results of haplotype analysis in patients groups and control group

Study groups	rs4359	rs1799752	Case	Control	Freq	OR (95%CI)	*P*-value	FDR q-Value (2)
OCD Patients	C	D	0.24	0.36	0.32	0.29 (0.19–0.45)	3.10E-09	6.26E-09
	C	I	0.34	0.28	0.30	1.76 (1.3–2.39)	2.30E-04	2.32E-04
	T	D	0.20	0.21	0.21	1.38 (0.99–1.91)	5.00E-02	3.37E-02
	T	I	0.21	0.13	0.15	0.99 (0.59–1.64)	9.70E-01	4.90E-01
SCZ Patients	C	D	0.27	0.36	0.33	0.44 (0.3–0.62)	2.50E-06	5.00E-06
	C	I	0.34	0.28	0.30	1.65 (1.25–2.2)	4.17E-04	4.21E-04
	T	D	0.15	0.21	0.20	1.31 (0.85–2.03)	2.12E-01	1.43E-01
	T	I	0.22	0.13	0.16	1.01 (0.73–1.38)	9.50E-01	4.80E-01
BPDI Patients	C	D	0.26	0.36	0.33	0.33 (0.21–0.51)	2.19E-07	4.42E-07
	C	I	0.30	0.28	0.29	1.61 (1.16–2.22)	4.00E-03	4.04E-03
	T	D	0.19	0.21	0.21	1.39 (0.99–1.97)	5.50E-02	3.70E-02
	T	I	0.23	0.13	0.15	1.04 (0.63–1.81)	7.80E-01	3.94E-01
BPDII Patients	C	D	0.24	0.36	0.32	0.33 (0.22–0.48)	1.98E-09	4.01E-09
	C	I	0.32	0.28	0.29	1.6 (1.2–2.13)	1.00E-03	1.01E-03
	T	D	0.17	0.21	0.20	1.57 (1.03–2.39)	3.40E-02	2.29E-02
	T	I	0.25	0.13	0.17	1.16 (0.85–1.58(	3.40E-01	1.72E-01

## Discussion

In the current study, we hypothesized that polymorphisms within *ACE* gene are associated with risk of some neuropsychiatric disorders. Our results verified this hypothesis. Renin-angiotensin system, particularly ACE enzyme has effective roles in the physiology of central nervous system. A previous meta-analysis has shown improvement in mental health domains of quality of life in patients that received angiotensin blockers/inhibitors for management of hypertension [21]. In addition, ACE activity has been associated with the etiopathogenesis of neuropsychiatric disorders including SCZ [22, 23]. Moreover, abnormalities in the DNA methylation pattern in *ACE* promoter has been identified as a causal factor for development of major depression [24].

Based on the functional importance of ACE in the regulation of blood pressure and its participation in modulation of risk of cardiovascular disorders, ACE might be involved in the potential bidirectional relation between mental and cardiovascular symptoms. This relation has been proposed for some years. In addition, recent surveys have confirmed the reported associations between depressive symptoms and cardiovascular disorders [25, 26].

Certain polymorphisms within *ACE* gene have been associated with risk of neuropsychiatric disorders. For instance, the functional SNP rs4291 has been shown to affect activity of hypothalamic-pituitary-adrenocortical system representing a mutual pathophysiologic connection for unipolar depression and cardiovascular disorder [27]. The rs1799752 polymorphism of this gene has been shown to be associated with panic disorder and principally respiratory type of this disorder. Moreover, this polymorphism can affect treatment outcomes of these patients. In fact, D allele of rs1799752 polymorphism has been linked with the severity of panic disorder [28].

In the current case–control study, we appraised the association between the rs4359 and rs1799752 polymorphisms and risk of BPD, SCZ and OCD. In allelic model, the effective I allele of rs1799752 showed a significant effect toward the risk for all patients groups. Previous studies have shown the impact of this polymorphism on expression of ACE in a way that the marker allele I has been linked with lower ACE levels [29]. Similarly, ACE levels have been shown to be highest among individuals having DD-genotype, followed by those having ID and II-genotypes, respectively [30].

The effective T allele of rs4359 showed a significant effect toward the risk for BPDII group. The rs4359 was associated with risk of OCD, BPDI and BPDII in co-dominant and dominant models. The rs1799752 was associated with all assessed psychiatric conditions in four inheritance models except for BPDII whose association was not significant in recessive model.

Cumulatively, the current data support involvement of two polymorphisms of *ACE* gene in conferring risk of diverse psychiatric disorders. Further assessment of expression levels of ACE in the circulation of these patients is necessary for confirming the association between these polymorphisms and risk of psychiatric disorders. Our study has some limitations such as lack of functional assays and small sample size which might influence the results of study, particularly in sex-based analyses.

## Supplementary Information

**Additional file 1:** 

**Additional file 2:** **Table S1.** The genotype and allele frequencies of thers4359 in different study groups, namely OCD (obsessive-compulsive disorder), SCZ (Schizophrenia), BPDI (bipolardisorder class I), BPDII (bipolar disorder class II) and NC (normal control) (*shows significance). **Table S2.** The genotype and allele frequencies of thers1799752 in study groups of OCD (obsessive-compulsive disorder), SCZ (Schizophrenia), BPDI (bipolardisorder class I), BPDII (bipolar disorder class II) and NC (normal control). **Table S3.** Associations between rs4359 polymorphism anddisorders were assessed in co-dominant, dominant, recessive and over-dominantmodels (*shows significance). **Table S4.** Associations between rs1799752 polymorphism and disorders were assessed inco-dominant, dominant, recessive and over-dominant models (*showssignificance).

## Data Availability

The datasets used and/or analyzed during the current study are available from the corresponding author on reasonable request.
