# Supplementary material for: Angiotensin I converting enzyme gene polymorphisms and risk of psychiatric disorders
Source: BMC Psychiatry. 2022 May 23;22:351. doi: 10.1186/s12888-022-04007-w (PMC9128292; doi:10.1186/s12888-022-04007-w)
Supplement: Supplementary file 2 — Additional file 2: Table S1. The genotype and allele frequencies of thers4359 in different study groups, namely OCD (obsessive-compulsive disorder), SCZ (Schizophrenia), BPDI (bipolardisorder class I), BPDII (bipolar disorder class II) and NC (normal control) (*shows significance). Table S2. The genotype and allele frequencies of thers1799752 in study groups of OCD (obsessive-compulsive disorder), SCZ (Schizophrenia), BPDI (bipolardisorder class I), BPDII (bipolar disorder class II) and NC (normal control). Table S3. Associations between rs4359 polymorphism anddisorders were assessed in co-dominant, dominant, recessive and over-dominantmodels (*shows significance). Table S4. Associations between rs1799752 polymorphism and disorders were assessed inco-dominant, dominant, recessive and over-dominant models (*showssignificance). [file 12888_2022_4007_MOESM2_ESM.docx]

Supplementary material

Table S1. The genotype and allele frequencies of the rs4359 in different study groups, namely OCD (obsessive-compulsive disorder), SCZ (Schizophrenia), BPDI (bipolar disorder class I), BPDII (bipolar disorder class II) and NC (normal control) (*shows significance).

| SNPs | Genotypes | | |  | Alleles | |  |
| --- | --- | --- | --- | --- | --- | --- | --- |
|  | T/T | T/C | C/C | χ2 P | T | C | χ2 P |
| NC  Males (n=236)  Females (n=80)  Total (n=316) | 31 (13.1)  14 )17.5)  45 (14.2) | 98 (41.5)  34 )42.5)  132 (41.8( | 107 (45.3)  32 )40)  139 (44) |  | 160 (33.9)  62 (38.8)  222 (35.1) | 312 (66.1)  98 (61.3)  410 (64.9) |  |
| OCD  Males (n=34)  Females (n=86)  Total (n=120) | 3 (8.8)  12 (14)  15 (12.5) | 21 (61.8(  49 (57)  70 (58.3) | 10 )29.4)  25 (29.1)  35 (29.2) | 4.9 0.08  3.5 0.17  10.1 0.006* | 27 (39.7)  73 (42.4)  100 (41.7) | 41 (60.3)  99 (57.6)  140 (58.3) | 0.88 0.34  0.46 0.49  3.14 0.07 |
| SCZ  Males (n=92)  Females (n=58)  Total (n=150) | 18 (19.6)  5 (8.6)  23 (15.3) | 33 (35.9)  36 (62.1)  69 (46) | 41 (44.6)  17 (29.3)  58 (38.7) | 2.37 0.3  5.5 0.06  1.18 0.55 | 69 (37.5)  46 (39.7)  115 (38.3) | 115 (62.5)  70 (60.3)  185 (61.7) | 0.75 0.38  0.023 0.88  0.9 0.34 |
| BPD I  Males (n=41)  Females (n=61)  Total (102) | 4 (9.8)  10 (16.4)  14 (13.7) | 24 (58.5)  35 (57.4)  59 (57.8) | 13 (31.7)  16 (26.2)  29 (28.4) | 4.1 0.12  3.5 0.17  9.01 0.011* | 32 (39)  55 (45.1)  87 (42.6) | 50 (61)  67 (54.9)  117 (57.4) | 0.81 0.36  1.14 0.28  3.74 0.053 |
| BPD II  Males (n=57)  Females (n=89)  Total (146) | 7 (12.3)  14 (15.7)  21 (14.4) | 33 (57.9)  50 (56.2)  83 (56.8) | 17 (29.8)  25 (28.1)  42 (28.8) | 5.38 0.068  3.43 0.179  10.78 0.005* | 47 (58.8)  78 (56.2)  125 (42.8) | 67 (41.2)  100 (43.8)  167 (57.2) | 2.15 0.14  0.89 0.34  5.02 0.025* |

T/T homozygous reference; T/C, heterozygous; C/C, homozygous mutant; T, wild allele; C, mutant allele (based on SNP database); OCD, Obsessive-compulsive disorder; SCZ, Schizophrenia; BPD, bipolar disorder and NC, normal control. Genotype and allele frequencies are shown in parentheses. χ2 test and P value are presented for diagnostic groups vs. control subjects. According to the SNP database, the wild allele for rs4359 is the T allele; however, the alleles T was the minor allele in this study and was considered as effect allele.

Table S2. The genotype and allele frequencies of the rs1799752 in study groups of OCD (obsessive-compulsive disorder), SCZ (Schizophrenia), BPDI (bipolar disorder class I), BPDII (bipolar disorder class II) and NC (normal control).

| SNPs | Genotypes | | |  | Alleles | |  |
| --- | --- | --- | --- | --- | --- | --- | --- |
|  | D/D | D/I | I/I | χ2 P | D | I | χ2 P |
| NC  Males (n=236)  Females (n=80) Total (n=316) | 84 (35.6)  30 )37.5)  114 (36.1 | 105 (44.5)  33 )41.3)  138 (43.7( | 47 (19.9)  17 )21.3)  64 (20.3) |  | 273 (57.8)  93 (58.1)  366 (57.9) | 199 (42.2)  67 (41.9)  266 (42.1) |  |
| OCD  Males (n=34)  Females (n=86)  Total (n=120) | 0 (0)  2 (2.3)  2 (1.7) | 32 (94.1(  71 (82.6)  103 (85.8) | 2 )5.9(  13 (15.1)  15 (12.5) | 29.7 3.43E-7*  38.7 3.85E-15*  69.5 7.85E-16* | 32 (47.1)  75 (43.6)  107 (44.6) | 36 (52.9)  97 (56.4)  133 (55.4) | 2.8 0.09  6.99 0.008*  12.45 0.0004* |
| SCZ  Males (n=92)  Females (n=58)  Total (n=150) | 0 (0)  2 (3.4)  2 (1.3) | 79 (85.9)  47 (81)  126 (84) | 13 (14.1)  9 (15.5)  22 (14.7) | 54.1 1.7E-12*  26.5 0.000002*  80.2 3.75E-18* | 78 (42.4)  51 (44)  129 (43) | 106 (57.6)  65 (56)  171 (57) | 5.4 0.02*  12.7 0.0003*  18.1 0.00002* |
| BPD I  Males (n=41)  Females (n=61)  Total (n=102) | 0 (0)  1 (1.6)  1 (1) | 41 (100)  51 (83.6)  92 (90.2) | 0 (0)  9 (14.8)  9 (8.8) | 43.1 4.2E-10*  31.4 1.4E-7*  70.6 4.6E-16* | 41 (50)  146 (51.8)  94 (46.1) | 41 (50)  136 (48.2)  110 (53.9) | 1.74 0.186  5.97 0.014* 8.72 0.003* |
| BPD II  Males (n=57)  Females (n=89)  Total (146) | 2 (3.5)  1 (1.1)  3 (2.1) | 44 (77.2)  73 (82)  117 (80.1) | 11 (19.3)  15 (16.9)  26 (17.8) | 25.7 0.000003*  41.9 7.627E-10*  70 6.285E-16* | 48 (42.1)  75 (42.1)  123 (42.1) | 66 (57.9)  103 (57.9)  169 (57.9) | 9.17 0.002* 8.6 0.003*  19.9 0.000008* |

D/D homozygous reference; D/I, heterozygous; I/I, homozygous mutant; D, wild allele; I, mutant allele (based on SNP database); OCD, Obsessive-compulsive disorder; SCZ, Schizophrenia; BPD, bipolar disorder (class I and II) and NC, normal control. Genotype and allele frequencies are shown in parentheses. χ2 test and P value are presented for diagnostic groups vs. control subjects. According to the SNP database, the wild type allele for rs1799752 is D (deletion). The allele I (insertion) was the minor alleles in this study and was considered as effect allele.

Table S3. Associations between rs4359 polymorphism and disorders were assessed in co-dominant, dominant, recessive and over-dominant models (*shows significance).

| Study groups | Models | Genotypes | Case number (%) | Control number (%) | OR (95% CI) (1) | p-Value (1) | FDR q-Value (1) | OR (95% CI) (2) | p-Value (2) | FDR q-Value (2) |
| --- | --- | --- | --- | --- | --- | --- | --- | --- | --- | --- |
| OCD | Co-dominant | TT vs. CC | 15 (12.5) | 45 (14.2) | 1.45 (1.15-1.84) | 2.00E-03* | 4.00E-03* | 1.43 (1.1-1.86) | 6.00E-03* | 1.82E-02* |
|  |  | TC vs. CC | 70 (58.3) | 132 (41.8) | 1.31 (0.97-1.78) | 7.60E-02 | 6.14E-02 | 1.21 (0.86-1.7) | 2.50E-01 | 3.15E-01 |
|  | Dominant | TT+TC vs. CC | 85 (70.8) | 177 (56) | 1.9 (1.21-2.99) | 5.00E-03* | 6.70E-03* | 1.76 (1.07-2.89) | 2.40E-02* | 4.85E-02* |
|  | Recessive | TT vs. TC+CC | 15 (12.5) | 45 (14.2) | 0.86 (0.46-1.6) | 6.30E-01 | 4.24E-01 | 0.72 (0.36-1.43) | 3.50E-01 | 3.54E-01 |
|  | Over dominant | TT+CC vs. TC | 50 (41.7) | 184 (58.2) | 0.51 (0.33-0.78) | 2.00E-03* | 4.00E-03* | 0.5 (0.31-0.81) | 5.00E-03* | 1.82E-02* |
| SCZ | Co-dominant | TT vs. CC | 23 (15.3) | 45 (14.2) | 1.11 (0.9-1.38) | 2.90E-01 | 4.70E-01 | 1.06 (0.87-1.34) | 4.50E-01 | 6.70E-01 |
|  |  | TC vs. CC | 69 (46) | 132 (41.8) | 1.13 (0.86-1.5) | 3.50E-01 | 4.70E-01 | 1.11 (0.84-1.47) | 4.40E-01 | 6.70E-01 |
|  | Dominant | TT+TC vs. CC | 92 (61.3) | 177 (56) | 1.24 (0.83-1.85) | 2.70E-01 | 4.70E-01 | 1.18 (0.79-1.77) | 4.00E-01 | 6.70E-01 |
|  | Recessive | TT vs. TC+CC | 23 (15.3) | 45 (14.2) | 1.09 (0.63-1.88) | 7.50E-01 | 7.50E-01 | 1.1 (0.64-1.9) | 7.10E-01 | 7.10E-01 |
|  | Over dominant | TT+CC vs. TC | 81 (54) | 184 (58.2) | 0.84 (0.56-1.24) | 3.90E-01 | 4.70E-01 | 0.89 (0.56-1.32) | 5.60E-01 | 6.70E-01 |
| BPD-I | Co-dominant | TT vs. CC | 14 (13.7) | 45 (14.2) | 1.46 (1.13-1.87) | 3.00E-03* | 1.20E-02* | 1.43 (1.1-1.86) | 8.00E-03* | 2.40E-02* |
|  |  | TC vs. CC | 59 (578) | 132 (41.8) | 1.36 (0.99-1.88) | 5.60E-02 | 6.70E-02 | 1.27 (0.9-1.78) | 1.66E-01 | 2.01E-01 |
|  | Dominant | TT+TC vs. CC | 73 (71.6) | 177 (56) | 1.97 (1.21-3.2) | 6.00E-03* | 1.20E-02* | 1.82 (1.1-3.03) | 1.90E-02* | 3.80E-02* |
|  | Recessive | TT vs. TC+CC | 14 (13.7) | 45 (14.2) | 0.95 (0.52-1.8) | 8.90E-01 | 8.90E-01 | 0.83 (0.42-1.64) | 8.30E-01 | 8.30E-01 |
|  | Over dominant | TT+CC vs. TC | 43 (42.2) | 184 (58.2) | 0.52 (0.33-0.82) | 5.00E-03* | 1.20E-02* | 0.52 (0.32-0.84) | 8.00E-03* | 2.40E-02* |
| BPD-II | Co-dominant | TT vs. CC | 21 (14.4) | 45 (14.2) | 1.44 (1.15-1-79) | 1.00E-03* | 1.00E-03* | 1.41 (1.12-1.79) | 3.00E-03* | 7.80E-03* |
|  |  | TC vs. CC | 83 (56.8) | 132 (41.8) | 1.38 (1.03-1.83) | 2.60E-02* | 5.00E-03* | 1.3 (0.96-1.76) | 8.80E-02 | 5.50E-02 |
|  | Dominant | TT+TC vs. CC | 104 (71.2) | 177 (56) | 1.94 (1.27-2.96) | 2.00E-03* | 1.00E-03* | 1.83 (1.17-2.85) | 8.00E-03* | 8.40E-03* |
|  | Recessive | TT vs. TC+CC | 21 (14.4) | 45 (14.2) | 1.01 (0.57-1.77) | 9.60E-01 | 1.68E-01 | 0.9 (0.49-1.63) | 7.30E-01 | 3.83E-01 |
|  | Over dominant | TT+CC vs. TC | 63 (43.2) | 184 (58.2) | 0.54 (0.36-0.81) | 3.00E-03* | 1.00E-03* | 0.54 (0.35-0.83) | 5.00E-03* | 7.80E-03* |

Table S4. Associations between rs1799752 polymorphism and disorders were assessed in co-dominant, dominant, recessive and over-dominant models (*shows significance).

| Study groups | Models | Genotypes | Case number (%) | Control number (%) | OR (95% CI) (1) | p-Value (1) | FDR q-Value (1) | OR (95% CI) (2) | p-Value (2) | FDR q-Value (2) |
| --- | --- | --- | --- | --- | --- | --- | --- | --- | --- | --- |
| OCD | Co-dominant | II vs. DD | 15 (12.5) | 64 (20.3) | 4.69 (3.05-7.22) | 2.04E-12* | 1.03E-12* | 5.26 (3.27-8.46) | 6.93E-12* | 3.50E-12* |
|  |  | ID vs. DD | 103 (85.8) | 138 (43.7) | 1.85 (1.33-2.57) | 2.22E-04* | 5.60E-05* | 1.88 (1.29-2.73) | 8.52E-04* | 2.15E-04* |
|  | Dominant | ID+II vs. DD | 118 (98.3) | 202 (63.9) | 33.29 (8.07-137.2) | 1.00E-06* | 3.37E-07* | 35.2 (8.3-148.3) | 1.00E-06* | 3.37E-07* |
|  | Recessive | II vs. ID+DD | 15 (12.5) | 64 (20.3) | 0.56 (0.3-1.03) | 6.30E-02 | 1.06E-02 | 0.5 (0.26-0.97) | 4.00E-02* | 6.73E-03* |
|  | Over dominant | II+DD vs. ID | 17 (14.2) | 178 (56.3) | 0.12 (0.07-0.22) | 5.74E-13* | 5.79E-13* | 0.11 (0.06-0.2) | 2.00E-12* | 2.02E-12* |
| SCZ | Co-dominant | II vs. DD | 22 (14.7) | 64 (20.3) | 4.45 (3.04-6.49) | 1.06E-14* | 9.91E-15* | 4.55 (3.1-6.68) | 9.59E-15* | 8.57E-15* |
|  |  | ID vs. DD | 126 (84) | 138 (43.7) | 2.01 (1.47-2.75) | 1.10E-05* | 3.00E-06* | 2.02 (1.47-2.78) | 1.20E-05* | 3.00E-06* |
|  | Dominant | ID+II vs. DD | 148 (98.7) | 202 (63.9) | 41.7 (10.15-171.7) | 2.30E-07* | 7.76E-08* | 42.7 (10.37-176.4) | 2.02E-07* | 6.81E-08* |
|  | Recessive | II vs. ID+DD | 22 (14.7) | 64 (20.3) | 0.67 (0.39-1.14) | 1.40E-01 | 2.36E-02* | 0.66 (0.39-1.14) | 1.39E-01 | 2.30E-02* |
|  | Over dominant | II+DD vs. ID | 24 (16) | 178 (56.3) | 0.148 (0.09-0.24) | 1.96E-14* | 9.91E+15* | 0.144 (0.08-0.23) | 1.70E-14* | 8.57E-15* |
| BPD-I | Co-dominant | II vs. DD | 9 (8.8) | 64 (20.3) | 6.28 (3.6-10.95) | 9.49E-11* | 2.99E-10* | 7.08 (3.91-12.8) | 9.02E-11* | 2.84E-10* |
|  |  | ID vs. DD | 92 (90.2) | 138 (43.7) | 1.72 (1.22-2.43) | 2.00E-03* | 3.10E-03* | 1.69 (1.17-2.44) | 5.00E-03* | 6.30E-03* |
|  | Dominant | ID+II vs. DD | 101 (99) | 202 (63.9) | 57 (7.8-414) | 6.40E-05* | 1.30E-04* | 59.2 (8-434) | 5.90E-05* | 1.24E-04* |
|  | Recessive | II vs. ID+DD | 9 (8.8) | 64 (20.3) | 0.38 (0.18-0.79) | 1.00E-02* | 1.00E-02* | 0.33 (0.155-71) | 5.00E-03* | 6.30E-03* |
|  | Over dominant | II+DD vs. ID | 10 (9.8) | 178 (56.3) | 0.084 (0.042-0.16) | 2.03E-12* | 1.28E-11* | 0.073 (0.036-0.15) | 1.22E-12* | 7.68E-12* |
| BPD-II | Co-dominant | II vs. DD | 26 (17.8) | 64 (20.3) | 3.72 (2.63-5.25) | 9.86E-14* | 1.04E-13* | 3.79 (2.63-5.46) | 8.43E-13* | 8.85E-13* |
|  |  | ID vs. DD | 117 (80.1) | 138 (43.7) | 2.08 (1.52-2.85) | 4.00E-06* | 1.00E-06* | 2.14 (1.53-3) | 9.00E-06* | 2.00E-06* |
|  | Dominant | ID+II vs. DD | 143 (97.9) | 202(63.9) | 26.9 (8.38-86.34) | 3.15E-08* | 1.10E-08* | 27.1 (8.32-88.29) | 4.32E-08* | 1.51E-08* |
|  | Recessive | II vs. ID+DD | 26 (17.8) | 64 (20.3) | 0.85 (0.51-1.4) | 5.30E-01 | 9.20E-02 | 0.85 (0.49-1.46) | 5.60E-01 | 9.80E-02 |
|  | Over dominant | II+DD vs. ID | 29 (19.9) | 178 (56.3) | 0.19 (0.12-0.3) | 3.02E-12* | 1.59E-12* | 0.19 (0.11-0.31) | 3.38E-11* | 1.78E-11* |
